# Pain in trigeminal neuralgia: neurophysiology and measurement: a comprehensive review

**Published:** 2013-12-25

**Authors:** S Kumar, S Rastogi, S Kumar, P Mahendra, M Bansal, L Chandra

**Affiliations:** * Private Practice; ** Department of Oral and Maxillofacial Surgery and Oral Implantology, Institute of Technology and Sciences- Centre for Dental Studies and Research, Murad Nagar, Ghaziabad, India-201206; *** Department of Periodontology, Institute of Dental Studies and Technologies, CCS University, Modinagar, Uttar Pradesh, India

**Keywords:** pain assessment, trigeminal neuralgia, neurophysiology, quantitative sensory testing

## Abstract

Abstract

Trigeminal neuralgia (TN) is defined as sudden, usually unilateral, severe, brief, stabbing recurrent episodes of pain within the distribution of one or more branches of the trigeminal nerve. It is the most frequent cranial neuralgia, the incidence being 1 per 1,000,00 persons per year. Pain attacks start abruptly and last several seconds but may persist 1 to 2 minutes. The attacks are initiated by non painful physical stimulation of specific areas (trigger points or zones) that are located ipsilateral to the pain. After each episode, there is usually a refractive period during which stimulation of the trigger zone will not induce the pain. According to the European Federation of Neurological Societies (EFNS) guidelines on neuropathic pain assessment and the American Academy of Neurology (AAN)-EFNS guidelines on TN management the neurophysiological recording of trigeminal reflexes represents the most useful and reliable test for the neurophysiological diagnosis of trigeminal pains. The present article discusses different techniques for investigation of the trigeminal system by which an accurate topographical diagnosis and profile of sensory fiber pathology can be determined. With the aid of neurophysiological recordings and quantitative sensory testing, it is possible to approach a mechanism-based classification of orofacial pain.

## Introduction

The term "pain" is defined as "an unpleasant sensory and emotional experience associated with actual or potential tissue damage, or described in terms of such damage" by the International Association for the Study of Pain [**1**]. Pain is always subjective. The majority of patients complaining of pain in the orofacial region have an identifiable physical cause for their pain [**2**]. In order to make a definitive diagnosis, it is often necessary to establish a list of possible differential diagnosis and then to systematically exclude each by a process of elimination through patient history, clinical examination, diagnostic tests and investigations [**3**]. Differentiating between the disorders usually can be facilitated by determining the location, stimulus and characteristics of the pain. Knowledge of the age and sex predilections of each disorder can also be helpful [**3**].

 Facial pain or headache may be caused by toothache [**4**], but in turn, it can also be mimicked by several forms of disorders, myofacial pain, TMJ disorder, migraine, cluster headache, atypical facial pain and trigeminal neuralgia [**5,6**], or may be due to otolaryngologic disease [**7**].

 Trigeminal neuralgia is the most common type of neuralgia, and it is limited to the distribution of one or more branches of the trigeminal nerve. It is characterized by unilateral pain attacks which are sharp, shooting, lancinating, electric shock-like, burning and excruciating in nature.

 Pain attacks start abruptly and last several seconds but may persist for 1 to 2 minutes [**8,9**]. The attacks are initiated by non-painful physical stimulation of specific areas (trigger points or zones) that are located ipsilateral to the pain. After each episode, there is usually a refractive period during which stimulation of the trigger zone will not induce the pain [**10**]. The frequency of attacks depends on the sensitivity and localization of the trigger area.

Pain attacks are typically accompanied by tic-like cramps of the facial muscles, therefore the description "tic douloureux". A tic is an involuntary contraction or spasm of the muscles. The pain symptoms may be categorized under the following:

 • Dysaesthesia (abnormal perception of pain)

 • Allodynia (due to a stimulus which does not normally provoke pain)

 • Hyperalgesia (an increased sensitivity to pain).

 Neurophysiology:

 The complexity of understanding orofacial pain may be related to the underlying neurophysiological mechanisms such as activation of peripheral receptors, alteration of the size of receptive fields, neurotransmitter release, transmission and projection of nociceptive information, and convergence of nociceptive afferents onto common central neurons [**11**]. Mechanisms involved in the pathogenesis of pain involve the communication of a multitude of neurotransmitters and neuromodulators, which may play a key role in the perception of and response to pain [**12**]. 

 Central excitatory effects resulting from ongoing peripheral nociceptive input may occur. Nociception refers to electrochemical impulses that are transmitted from the periphery and are interpreted as pain in the upper brain. Continuous and/or recurrent nociceptive input into the central nervous system (CNS) may promote the release of neurotransmitter and vasoactive substances in the spinal trigeminal nucleus (subnucleus caudalis) [**13,14**]. This central excitation may decrease the threshold of adjacent second order neurons that receive input from sites other than nociceptive sources. As signals on excited second order neurons are transmitted to the higher centres (thalamus, limbic system, and somatosensory cortex), nociception is interpreted as pain [**15**].

**Fig. 1:**
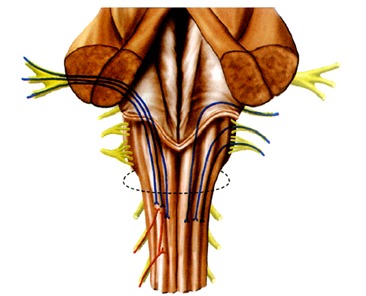
Convergence of primary small fibres subserving nociception

 The trigeminal convergence-projection theory (**Fig. 1**) is based on the fact that nociceptive input from virtually the entire head and neck (cranial nerves V, VII, IX and X and cervical nerves 1-4) converge on the trigeminal spinal nucleus (subnucleus caudalis) [**16-18**]. This area is where primary nociceptive nerves synapse with second order neurons. This concept suggests that it is in this area where central excitation occurs. The number of primary pain- transmitting neurons is far greater than the number of second order neurons. Therefore, sensory input from multiple regions, including the cervical region, may project onto the same second order neuron for transmission to the higher centres in the pain pathways where pain localization, interpretation and response occur.

**Fig. 2:**
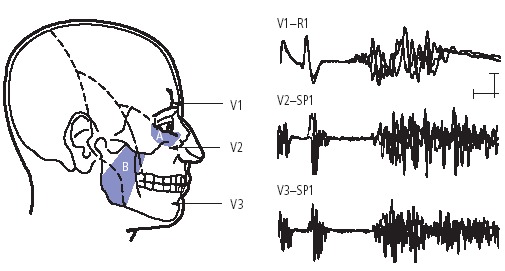
Trigeminal reflexes for diagnosing symptomatic trigeminal neuralgia One early response is measured for each of the three trigeminal divisions: the R1 blink reflex after stimulation of the supraorbital nerve (V1-R1), the SP1 masseter inhibitory reflex after stimulation of the infraorbital nerve (V2-SP1) and that after stimulation of the mental nerve (V3-SP1). Figure shows surface recordings from the orbicularis oculi muscle (for V1) and masseter muscle for (V2 and V3).

 Neurophysiological investigations:

According to the European Federation of Neurological Societies (EFNS) guidelines on neuropathic pain assessment and the American Academy of Neurology (AAN)-EFNS guidelines on trigeminal neuralgia management [**19,20**], the neurophysiological recording of trigeminal reflexes represents the most useful and reliable test for the neurophysiological diagnosis of trigeminal pains. The trigeminal reflexes (**Fig. 2**) consist of a series of reflex responses (R1 and R2 components of the blink reflex after electrical stimulation of the ophthalmic division; SP1 and SP2 components of the masseter inhibitory reflex after electrical stimulation of the maxillary or mandibular division) that assess function of trigeminal afferents from the three trigeminal divisions, as well as trigeminal central circuits in the brainstem.

 In patients reporting with pain in the trigeminal territory, trigeminal reflexes offer the clinician with useful information. Abnormalities are often discovered in divisions that appear clinically unaffected. An objective demonstration of dysfunction is provided in all patients with pain secondary to a documented disease, such as symptomatic trigeminal neuralgia, post-herpetic neuralgia, vascular malformations, benign tumors of the cerebellopontine angle and multiple sclerosis. As a tool for disclosing symptomatic trigeminal neuralgia, neurophysiological testing of trigeminal reflexes provides the same sensitivity (95%) and specificity (93%) as MRI [**21**].

 Measurement of pain:

 A wide range of treatment options exists for this disorder, including medical management, microvascular decompression, radiosurgery, percutaneous trigeminal ganglion techniques, etc. Any attempt to compare these modalities or to develop effective new therapies requires the availability of reliable and validated pain scales. However, chronic pain in trigeminal neuralgia and related facial pain syndromes is difficult to measure because of its subjective nature and the strong influence of social context, emotions and other non-physiological variables. In the neurological literature, the most common approach to evaluating this type of pain has used measurements of pain intensity or percent pain relief. These one-dimensional instruments do not adequately address the complexity of the measurement of chronic pain, and they have not undergone psychometric testing to assess their reliability and validity in trigeminal neuralgia patients.

**Fig. 3:**
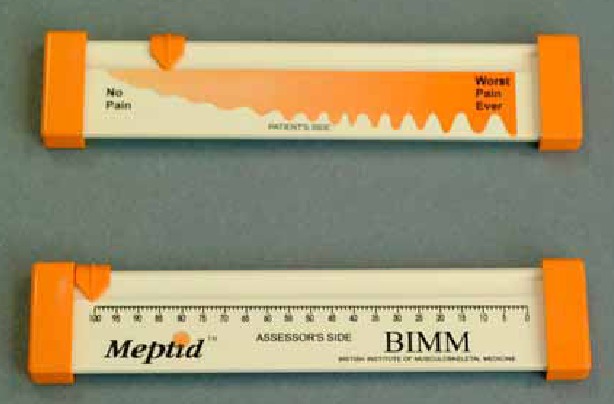
Visual Analog Scale

**Fig. 4:**
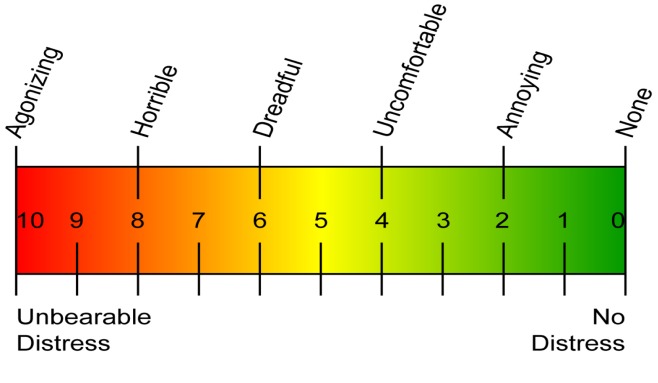
Visual Analog Scale

 Current trigeminal neuralgia pain scales:

 Pain scales in the neurological literature have typically relied on a single measure of pain intensity or a composite scale of pain intensity and medication use. The prototypical assessment of pain intensity is the Visual Analog Scale (VAS) [**22**]. This instrument consists of a 10 cm (100 mm) line with verbal anchors at each end (e.g.- 0 representing "no pain" and 100 representing "worst pain"). Patients mark on this line the point which they feel best represents their perception of pain (**Fig. 3,Fig. 4**). It is a continuous scale that allows the estimation of pain intensity. As line length in VAS is a response continuum, many patients find it difficult to judge the distance accurately. Hence, this can also be used as a verbal rating scale asking patients to express their pain as range from 0 to 10. VAS scoring has linear scoring properties (i.e. the difference in pain between each successive increment equals to 10) [**22**]. Thus, a VAS pain score of 60 mm indicates twice as much pain as a VAS score of 30 mm. Also, the difference between the VAS score of 30 and 40 mm would be of the same magnitude as the difference between the VAS scores of 70 and 80 mm.

 Over decades of use, the VAS has been demonstrated to be a sensitive, reproducible pain scale in rheumatologic disorders [**22**], cancer [**23**], degenerative joint disease [**24**] and other disease processes. In trigeminal neuralgia, the VAS has been used to determine the efficacy of medical therapies [25-27], as well as surgical interventions such as microvascular decompression [**28**] and motor cortex stimulation [**29**]. VAS is a reproducible measure of a single facet of pain, i.e., pain intensity at the time at which the patient completes the survey. An evaluation of various length and end-phrase variations of VAS showed that the 10 cm VAS had the smallest measurement error, while the end-phrase "worst pain imaginable" had the greatest sensitivity in measuring "present pain" for acute dental pain [**30**].

Composite scales in the neurological literature on trigeminal neuralgia have also been used [**31**]. These include 2 elements. The first part often involves a measure of pain intensity in 3 to 5 categories specifying the level of pain (e.g. none, some and severe). Alternatively, another method used in some studies is the "global assessment in change", which is usually expressed as a percentage decrease in pain [**32**]. The second part of these composite scales describes the level of medication usage such as no medication use, reduced medication use and continued medication use. The classification scheme devised by the group at the Barrow Neurological Institute (BNI) has become widely used and is the prototypical composite scale (**Table 1**). The BNI score is compared at subsequent visits of the patient and is also used for comparison within two or more patients. Composite scales have been useful because they have allowed some degree of standardization across treatment modalities, but their reliability and validity have not been tested thoroughly.

**Table 1:**
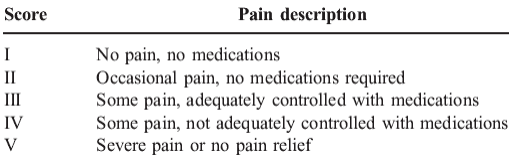
Barrow Neurological Institute Pain Intensity Score

Another commonly used pain assessment tool is the McGill Pain Questionnaire (MPQ) which allows the patient to indicate easily the quality of his/her pain using such descriptors as throbbing, shooting, distressing, excruciating etc. [**33**]. It contains a total of 78 words (pain descriptors) which can assess sensory and affective pain qualities (**Table 2**). The descriptors fall into four major groups:

 • Sensory – 1 to 10

 • Affective – 11 to 15

 • Evaluative -16

 • Miscellaneous – 17 to 20.

 The patient is asked to circle a word that best describes his pain in each word set. The rank value for each descriptor is based on its position in the word set [e.g. – flickering(1), quivering(2), pulsing(3), throbbing(4), beating(5), pounding(6), jumping(1), flashing(2), shooting(3) etc. The sum of the rank values is the Pain Rating Index (PRI). Minimum PRI is 0 and maximum PRI is 78. PRI can be compared at subsequent visits of the patient and is also used for comparison within two or more patients.

**Table 2:**
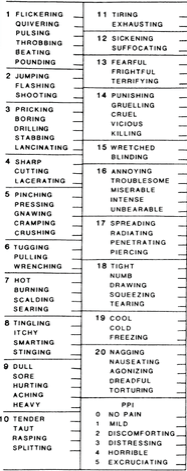
McGill Pain Questionnaire

 Only one study, however, has used the MPQ for analyzing chronic orofacial pain conditions, with the instrument correctly predicting the diagnosis in 90 percent of patients with trigeminal neuralgia [**34**]. Patients may be reluctant to fill out multipage questionnaires and sometimes even resort to ‘satisfying’ responses simply to complete the survey.

 New trigeminal neuralgia pain scales:

 More comprehensive approaches to evaluating pain have begun to be applied in trigeminal neuralgia. The multi-institutional Initiative on Methods, Measurement and Pain Assessment in Clinical Trials (IMMPACT) had defined 6 core domains to be considered in treatment trials of chronic pain:

 1) pain intensity

 2) physical functioning

 3) emotional functioning

 4) participant ratings of improvement and satisfaction with treatment

5) symptoms and adverse effects

 6) participant adherence to treatment regimens [**35**].

 Specific instruments have been recommended for outcome measurements with these domains in mind (**Table 3**).

**Table 3:**
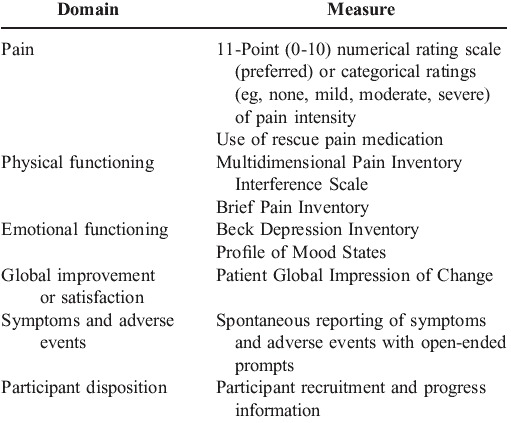
Initiative on Methods, Measurement and Pain Assessment in Clinical Trials Recommendations for Core Outcome Measures

**Fig. 5:**
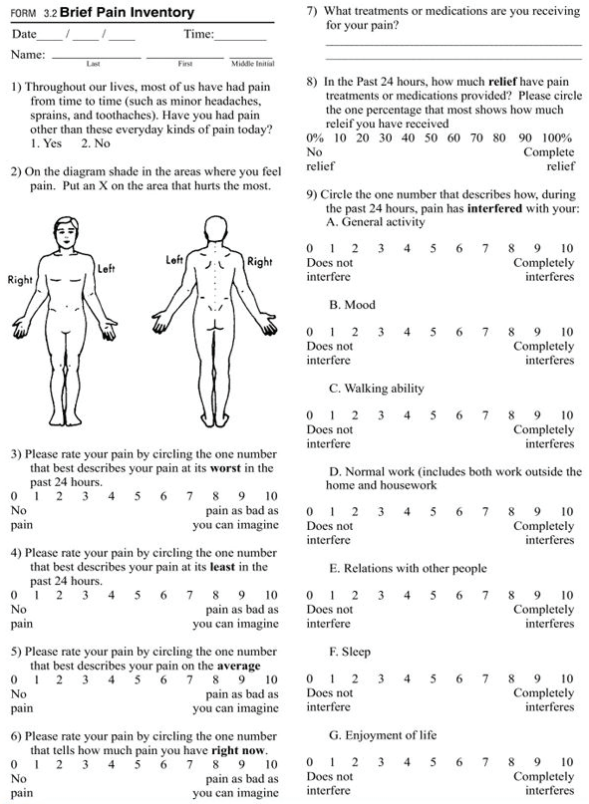
Brief Pain Inventory Scale

 The ideal pain scale should effectively examine several domains of pain in a time-efficient manner. Based on the IMMPACT recommendations, the Brief Pain Inventory BPI-Facial scale was developed which concentrates on the first 2 domains of the IMMPACT recommendations [**36**]. This instrument is composed of 18 items on a 1-point scale (0-10). 4 questions centre on pain intensity, 7 questions deal with the interference of pain with general life activities and the remaining 7 questions deal with the interference of pain with face-specific activities (**Table 4,Fig. 5**).

**Table 4:**
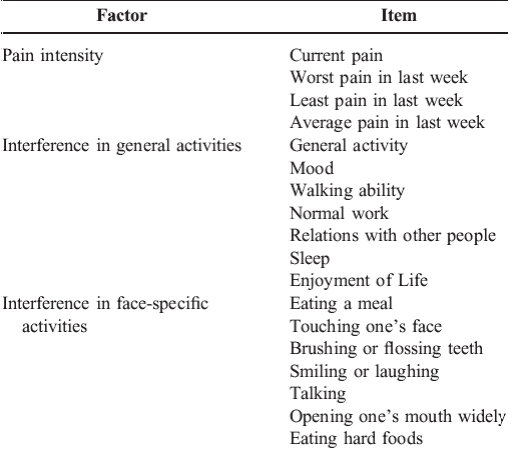
BPI-Facial scale

The patient is asked to rate the severity of his pain on a scale of 0-10 at different specified time intervals. The patient is also required to represent the location of his pain on a schematic diagram of the body. The patient is asked to mark on a scale of 0-10 the amount of interference of pain with 7 listed general life activities and 7 listed face-specific activities. The mean is calculated for both of these. The values obtained can be used for comparison at different time intervals for the same patient as well as for comparison between different patients.

## Conclusion 

 Although more advanced techniques assessing brain responses after selective nociceptive stimulation provide important pathophysiological information, a diagnostic protocol for patients with trigeminal pain should rely primarily on trigeminal reflexes. The technique is easy and completely harmless and the finding of any abnormality implies an underlying structural lesion. It can be easily performed using an electromyography unit. It also has the advantage of being far less expensive than MRI.

 Along with the pathophysiology of pain, the accurate measurement of pain is also crucial for determining the efficacy of surgical and medical therapies. Although neurosurgical studies on trigeminal neuralgia traditionally have not used pain measurement tools, there have been some recent attempts to quantify pain using various pain measurement scales, recommendations of the IMMPACT consortium, BPI-Facial scale, etc. However, to quantify such pain in a time-efficient manner, we still need thoughtfully constructed instruments with proven reliability and validity.
